# New synthetic cannabinoids and the potential for cardiac arrhythmia risk

**DOI:** 10.1016/j.jmccpl.2023.100049

**Published:** 2023-12

**Authors:** Jules C. Hancox, Caroline S. Copeland, Stephen C. Harmer, Graeme Henderson

**Affiliations:** a School of Physiology, Pharmacology and Neuroscience, Biomedical Sciences Building, University Walk, Bristol BS8 1TD, UK; b Institute of Pharmaceutical Science, King's College London, UK; c Centre for Pharmaceutical Medicine Research, King's College London, UK

**Keywords:** Cannabis, Synthetic cannabinoid receptor agonists (SCRAs), hERG, Long QT, Torsades de pointes

## Abstract

Synthetic cannabinoid receptor agonists (SCRAs) have been associated with QT interval prolongation. Limited preclinical information on SCRA effects on cardiac electrogenesis results from the rapid emergence of new compounds and restricted research availability. We used two machine-learning-based tools to evaluate seven novel SCRAs' interaction potential with the hERG potassium channel, an important drug antitarget. Five SCRAs were predicted to have the ability to block the hERG channel by both prediction tools; ADB-FUBIATA was predicted to be a strong hERG blocker. ADB-5Br-INACA and ADB-4en-PINACA showed varied predictions. These findings highlight potentially proarrhythmic hERG block by novel SCRAs, necessitating detailed safety evaluations.

## Highlights

• Synthetic cannabinoid receptor agonists (SCRAs) are illicitly used psychotropics.
• Mounting evidence suggests that SCRAs can affect cardiac repolarisation.
• Two *in silico* tools were used to evaluate whether 7 novel SCRAs may inhibit “hERG”.
• 5 of the 7 SCRAs were predicted by both tools to inhibit the hERG potassium channel.
• These results highlight a potential route for adverse cardiac effects of SCRAs.

## Graphical abstract

**Figure:**
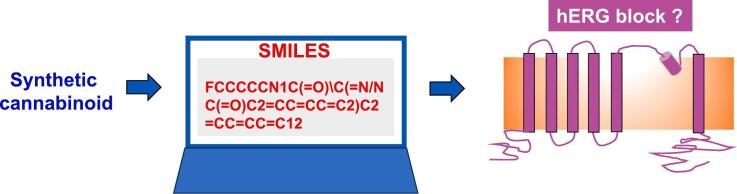

## 1 Introduction

A 2021 analysis of drug-related deaths reported to the UK National Programme on Substance Abuse Deaths (NPSAD) for England (census period 2012–2019) highlighted 165 deaths linked to the use of synthetic cannabinoid receptor agonists (SCRAs) [1], psychotropic drugs, most of which have no clinical indication. Approximately 75 % of these deaths were linked to cardiorespiratory complications and of those that were witnessed, ∼82 % were described as having involved sudden collapse [1]. There is growing evidence of a link between SCRA use and risk of cardiac arrhythmia [2]. Case reports and patient cohort studies have highlighted the potential for SCRAs to produce QT interval changes on the electrocardiogram (ECG) reminiscent of those observed in drug-induced long QT syndrome (diLQTS) [[3], [4], [5], [6], [7], [8], [9]]. diLQTS predisposes to the arrhythmia *torsades de pointes* (TdP). Therefore, administration of drugs with a propensity for QT-prolongation should be undertaken with caution in the clinical management of SCRA users [2].

One recent study of 72 SCRA users, of whom 68 were males, has reported P wave and QT interval abnormalities (including statistically significant increases in both rate-corrected QT interval duration and QT dispersion) in comparison with 27 age and sex matched controls (22 males) [8]. In multivariate analysis, serum potassium was found to be an independent predictor of premature ventricular beats [8]. It is well established that electrolyte abnormalities, including hypokalemia, are strongly associated with diLQTS and TdP [10]. In a 2022 study of 41 male SCRA users and a similar number of age-matched healthy controls, heart rate did not differ between the groups but SCRA users exhibited P wave dispersion, and increased QT interval, rate corrected QT (QT_c_) interval, QT dispersion and T_peak_-T_end_ values compared to controls [9]. The changes to markers of ventricular repolarization in this study are in good agreement with those reported in a prior study of 58 SCRA-using hospital patients, who exhibited longer QT_c_ and T_peak_-T_end_ values than from 50 healthy controls [5]. Thus, the available clinical literature provides consistent evidence that SCRA use is linked to delayed ventricular repolarization.

Despite this, there remains relatively little preclinical literature on the underlying basis of altered ventricular repolarization with SCRAs. Cannabidiol, which is structurally distinct from SCRAs, has been shown to inhibit the “hERG” (Kv11.1) potassium channel and prolong ventricular action potential duration at some concentrations [11]. hERG block is the major culprit in diLQTS and novel pharmaceuticals are routinely evaluated for any propensity to inhibit hERG channels. One SCRA study has reported JWH-030 to act as a relatively low affinity inhibitor of hERG [12], but data on other SCRAs are lacking.

Street preparations of SCRAs may contain unidentified SCRAs or SCRA mixtures, and patterns of SCRA use continually evolve as new compounds emerge [1]. This complicates preclinical mechanistic analysis of SCRA toxicology. This problem is well illustrated by the diversity of SCRAs highlighted by the United Nations Office on Drugs and Crime (UNODC), which recently noted that 320 SCRAs had been reported to the UNODC early warning advisory on new psychoactive substances (UNODC-EWA) since 2009. In May of 2022, the UNODC reported detection of seven new unique SCRAs found in East and South-East Asia for the first time in 2021. Four of these are from the new “OXIZID” (*N*-alkylisatin-acylhydrazone) group, which may result from efforts to circumvent legal controls. The advisory from the UNODC reporting the emergence of these compounds highlights that most OXIZID analogues are unstudied with undetermined pharmacological effects.

The gold-standard approach to evaluating drugs for delayed ventricular repolarization involves patch-clamp evaluation of hERG channel inhibition *in vitro*. However, the near ubiquity of hERG block by QT interval prolonging drugs has led to efforts towards *in silico* screens that can be employed early in the evaluation process. This approach is well suited to the initial evaluation of novel compounds of restricted availability. Here we employed two publicly accessible online computational tools “PredhERG” and “hERGSPred” to evaluate the 7 new SCRAs recently reported by the UNODC.

## 2 Methods

**Fig. 1:**
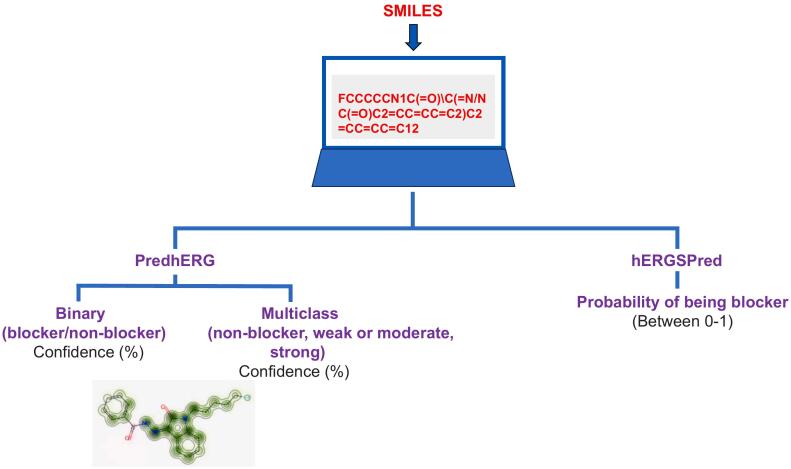
Schematic diagram illustrating prediction approaches used. Between December 2022 and April 2023, SMILES (Simplified Molecular Input Line Entry System) structures (such as that shown for 5F-BZO-POXIZID) for each compound evaluated were entered into the online user interface of the two evaluation tools. For compounds within its applicability domain (AD) PredhERG [13] provided binary (blocker/non-blocker) and multiclass (non-blocker, weak-moderate or strong blocker) predictions (see Table 1). The reported correct classification rates are 0.83–0.84 for the binary model and an accuracy of 0.66–0.79 for the multiclass model [13]. PredhERG also provides a visual output (example shown for 5F-BZO-POXIZID) highlighting in green molecular fragments recognized by the software that are likely to contribute to hERG block [13]. hERGSPred does not provide AD information and provides a non-synonymous output to PredhERG, evaluating the probability of a compound being a hERG blocker (output values between 0 and 1; see Table 1) using a consensus model with an accuracy of 0.839 [14].

PredhERG was developed from a curated hERG dataset of 5984 compounds and provides binary (blocker/non-blocker) and multiclass (non-blocker, weak-moderate or strong blocker) predictions with correct classification rates of 0.83–0.84 for the binary model and accuracy of 0.66–0.79 for the multiclass model [13]. hERGSPred utilised machine-learning-based models trained on 12,850 compounds to produce a consensus model with an accuracy of 0.839 [14]. PredhERG but not hERGSPred includes as part of its data output whether or not a chemical structure falls within the applicability domain (AD) of the model. Both tools have web-based user interfaces and when this study was conducted (between December 2022 and April 2023) were freely available for online use (http://predherg.labmol.com.br/ and http://www.icdrug.com/ICDrug/T). SMILES structures of 4 OXIZID-based SCRAs (5F-BZO-POXIZID, BZO-HEXOXIZID, BZO-POXIZID, BZO-CHMOXIZID) and 3 additional newly-emerged SCRAs (ADB-FUBIATA, ADB-5Br-INACA, ADB-4en-PINACA) were entered into each tool. For comparative purposes, two distinct positive control drugs were used: E-4031 (a known high potency hERG blocker (eg [15])) and the psychotropic drug escitalopram (an intermediate potency hERG blocker [16]). Atenolol was included as a non-hERG blocker negative control. Fig. 1 provides a schematic representation of the workflow involved in using these tools. Ventricular action potential simulations were conducted at 1 Hz using the open source “AP Predict” online cardiac electrophysiology simulator.

## 3 Results and discussion

Table 1 shows the prediction outcomes for the 7 new SCRAs. All 4 OXIZID compounds were rated by PredhERG to be potentially cardiotoxic with weak to moderate hERG block, whilst hERGSPred rated them as having high probabilities (from 0.98 to 0.99) of being hERG inhibitors. ADB-FUBIATA was predicted by PredhERG to be cardiotoxic with strong/extreme hERG block and by hERGSPred to be a hERG inhibitor with a probability of 0.95. There was less agreement between the two methods for ADB-5Br-INACA and ADB-4en-PINACA: these were found to be non-inhibitors by PredhERG, but to be inhibitors by hERGSPred with probabilities of 0.75 and 0.78 respectively. However, ADB-5Br-INACA was reported not to be within the AD of PredhERG, which confounds interpretation of the negative PredhERG result for this compound. All other structures were reported to lie within the AD for PredhERG.

**Table 1:** Predicted hERG channel inhibition for 7 recently identified novel SCRAs. SMILES structures for each of the molecules from the UNODC (linked to each SCRA molecule in left column) were input into “PredhERG” and “hERGSPred” web-based prediction models between December 2022 and April 2023. IUPAC structure names are included together with the prediction outcomes from each of the two prediction tools.

Compound	IUPAC name	PredhERG outcome (% confidence)	hERGSPred: probability of hERG interaction
5F-MDA-19 (5F-BZO-P**OXIZID**)	(*Z*)-*N*′-(1-(5-Fluoropentyl)-2-oxoindolin-3-ylidene)benzohydrazide	Potential cardiotoxic 50 % Weak or moderate 60 % Applicability domain Yes	0.99
MDA-19 (BZO-HEX**OXIZID**)	(*Z*)-*N*′-(1-Hexyl-2-oxoindolin-3-ylidene)benzohydrazide	Potential cardiotoxic 50 % Weak or moderate 60 % Applicability domain Yes	0.99
BZO-P**OXIZID**	(Z)-*N*′-(2-Oxo-1-pentylindolin-3-ylidene)benzohydrazide	Potential cardiotoxic 50 % Weak or moderate 60 % Applicability domain Yes	0.99
BZO-CHM**OXIZID**	(Z)-*N*′-(1-(Cyclohexylmethyl)-2-oxoindolin-3-ylidene)benzohydrazide	Potential cardiotoxic 50 % Weak or moderate 60 % Applicability domain Yes	0.98
ADB-FUBIATA	2-(2-(1-(4-Fluorobenzyl)-1H-indol-3-yl)acetamido)-3,3-dimethylbutanamide	Potential cardiotoxic 60 % Strong or extreme 50 % Applicability domain Yes	0.95
ADB-5Br-INACA	*N*-(1-Amino-3,3-dimethyl-1-oxobutan-2-yl)-5-bromo-1H-indazole-3-carboxamide	Non cardiotoxic 60 % Applicability domain No	0.75
ADB-4en-PINACA	*N*-(1-Amino-3,3-dimethyl-1-oxobutan-2-yl)-1-(pent-4-en-1-yl)-1H-indazole-3-carboxamide	Non cardiotoxic 50 % Applicability domain Yes	0.78
E-4031 (positive control)	*N*-[4-[1-[2-(6-Methylpyridin-2-yl)ethyl]piperidine-4‑carbonyl]phenyl]	Potential cardiotoxic 90 % Strong or extreme 70 % Applicability domain Yes	0.99
Escitalopram (positive control)	(1*S*)-1-[3-(Dimethylamino)propyl]-1-(4-fluorophenyl)-3*H*-2-benzofuran-5‑carbonitrile	Potential cardiotoxic 80 % Strong or extreme 50 % Applicability domain Yes	0.99
Atenolol (negative control)	2-[4-[2-Hydroxy-3-(propan-2-ylamino)propoxy]phenyl]acetamide	Non cardiotoxic 60 % Applicability domain Yes	0.12

**Fig. 2:**
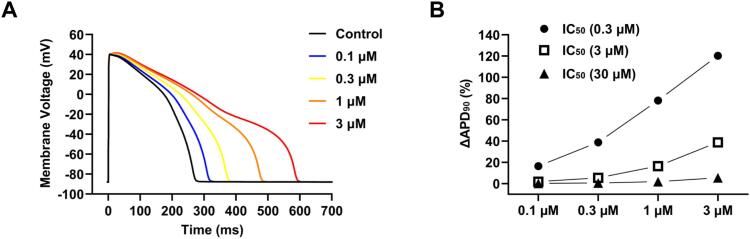
Illustrative prediction of action potential (AP) prolongation for different potency hERG blocking compounds. Ventricular AP simulations were conducted in August 2023 using the “AP Predict” online cardiac electrophysiology simulator [18]. From within the simulator the O'Hara Rudy CiPA ventricular AP model was selected and pIC_50_ values corresponding to strong/extreme (6.523), weak/moderate (5.523) and non-blocker (4.523) predicted inhibitory ranges of PredhERG were entered. APs were elicited at 1 Hz and for each pIC_50_ effects on APD_90_ of 0.1, 0.3, 1 and 3 μM were evaluated. (**A**) shows APs in control and at each inhibitor concentration, for a pIC_50_ of 6.523 (IC_50_ of 0.3 μM). (**B**) shows a plot of extent of prolongation of APD_90_ (ΔAPD_90_ (%)) at each test concentration for the 3 simulated different potency inhibitors.

The positive control E-4031 was considered by PredhERG as cardiotoxic with strong/extreme block, while hERGSPred gave a hERG inhibition probability of 0.99. Escitalopram had similar prediction outcomes (Table 1). The negative control atenolol was reported by PredhERG to be noncardiotoxic and hERGSPred gave a hERG inhibition probability of only 0.12. Neither of the prediction software suite versions employed here provided quantitative estimates of hERG channel/current blocking potency, though the multiclass model classifications of PredhERG are linked to concentration ranges: non-blockers with hERG activity at greater than or equal to 10 μM; weak or moderate blockers with hERG activity between 1 and 10 μM; strong or extreme blockers with hERG activity below or equal to 1 μM [13]. Comparative plasma level data for users of the SCRAs studied here were not available to us and, additionally, lipophilic compounds may accumulate in tissue. For illustrative purposes, we selected half-maximal inhibitory concentration (IC_50_) values within the 3 ranges given above: 300 nM, 3 μM and 30 μM (equivalent to pIC_50_ values of 6.523, 5.523, 4.523) and simulated effects on ventricular action potential (AP) repolarisation of 4 concentrations (0.1, 0.3, 1 and 3 μM) of an inhibitor with each of these IC_50_ values. Fig. 2A shows APs in control and each of the 4 inhibitor concentrations, for an IC_50_ of 0.3 μM (corresponding to the ‘strong or extreme’ condition). Fig. 2B contains plots of change (increase) in the AP duration at 90 % repolarisation (APD_90_) at each concentration tested, for the 3 IC_50_ values. The results predict relatively less extensive AP prolongation for weak or moderate hERG inhibition across the tested range (ΔAPD_90_ of 2–39 %) than the marked prolongation (ΔAPD_90_ of 16–120 %) predicted for strong or extreme hERG inhibition.

A limitation of the present study is that the methods employed here focused solely on hERG. While hERG is the main culprit in diLQTS, it is possible that SCRAs may affect other ion channels or electrogenic transporters, as recently reported for cannabidiol [17]. To our knowledge, hERG electrophysiology data on SCRA actions have not been reported except for JWH-030 [12]*.* Although *in silico* predictions cannot wholly substitute for experimental data, measured conclusions can be drawn from our findings. First, where new recreational compounds emerge faster than the ability to conduct detailed experimental safety/toxicology analyses, the use of these web-based *in silico* tools may provide potentially useful early insights. Second, the new OXIZID-based SCRAs seem likely to exert direct actions on the hERG channel, whilst the similar *in silico* results across the four compounds suggest that one or two OXIZID molecules may be selected for *in vitro* experimental investigation of what may be a class effect. Third, the strongest result was obtained for ADB-FUBIATA and this observation should be pursued further through direct investigation of hERG blockade and associated cardiotoxicity. Fourth, the more equivocal findings for ADB-5Br-INACA and ADB-4en-PINACA indicate caution in ascribing cardiotoxic potential to these compounds in the absence of *in vitro* data. Finally, that caution in using licensed medications with potential for diLQTS is likely warranted in people who use SCRAs. Overall, the results of this study underscore a need for comprehensive preclinical data on cardiac effects of SCRAs, in order to understand the link between these illicit substances and abnormal ventricular repolarization.

## Declaration of generative AI and AI assisted technologies in the writing process

During the preparation of this work the authors used ChatGTP 3.5 (Open AI) as part of the process of editing the Abstract. After using this tool the authors reviewed and edited the content as needed and take full responsibility for the content of the publication.

## Declaration of competing interest

The authors declare that they have no known competing financial interests or personal relationships that could have appeared to influence the work reported in this paper.
